# Unraveling the therapeutic potential of GANT61/Dactolisib combination as a novel prostate cancer modality

**DOI:** 10.1007/s12032-022-01718-8

**Published:** 2022-07-14

**Authors:** Mohamed Youssef, Nermine Moussa, Maged W. Helmy, Medhat Haroun

**Affiliations:** 1 Present Address: Department of Biotechnology, Institute of Graduate Studies and Research, Alexandria University, Alexandria, Egypt; 2 Department of Pharmacology & Toxicology, Faculty of Pharmacy, Damanhur University, Damanhur, Egypt

**Keywords:** PC3 cells, PI3K/AKT/mTOR, Hh/GLI, Dactolisib, GANT61

## Abstract

Aberrant activation of several signaling pathways has been implicated in prostate cancer (PCa) progression to castrate-resistant prostate cancer (CRPC). Phosphoinositide-3-kinase/Protein Kinase B/mechanistic Target of Rapamycin (PI3K/AKT/mTOR) and Hedgehog/GLI (Hh/GLI) pathways are major participants in progression to CRPC. In this sense, the current work aims to assess the potential antitumor effects resulting from co-targeting the aforementioned pathways in PC3 cells with Dactolisib as a dual PI3K/mTOR inhibitor and GANT61 as a *GLI1* antagonist. Three replica of PC3 cells were assigned for four treatment groups; vehicle control, Dactolisib-treated, GANT61-treated, and combination-treated groups. *GLI1* gene expression was determined by quantitative real-time PCR while active caspase-3 was determined colorimetrically. P-AKT, p70 ribosomal s6 protein kinase 1 (pS6K1), cyclin D1, vascular endothelial growth factor 1 (VEGF1), and Microtubule-associated proteins 1A/1B light chain 3 (LC3) protein levels were determined by ELISA technique. *GLI1* gene expression was down-regulated as a result of Dactolisib, GANT61, and their combination. Additionally, both drugs significantly reduced p-AKT, pS6K1, cyclin D1, and VEGF1 protein levels. Dactolisib elevated LC3 protein levels and GANT61 augmented Dactolisib effect on LC3. Moreover, only Dactolisib/GANT61combination significantly increased active caspase-3 level. To sum up, Dactolisib/GANT61 combination was shown to be promising in PCa treatment. Further *in-vitro* and *in-vivo* studies are warranted to support our findings.

## Introduction

According to GLOBOCAN 2018, prostate cancer (PCa) is the second most common cancer in men and the fifth leading cause of cancer death in men worldwide. Due to increased prostate-specific antigen testing practice, PCa incidence rates are higher in developed than in developing countries. The most advanced state of such disease is the so called castrate-resistant prostate cancer (CRPC) that seems to utilize endogenous androgen and other signaling pathways to proceed in its proliferation despite various therapeutic modalities [1].

Many studies have demonstrated that continuous proliferation of PCa cells and their resistance to traditional therapies may be attributed to inappropriate functioning of several genes and/or signaling pathways [2–4]. Androgen receptor*,* Forkhead box A1*,* Phosphatase and Tensin homolog*,* Retinoblastoma and NK3 Homeobox are among the genes implicated in prostate hyperplastic proliferation [4] while PI3K/AKT/mTOR [5] and Hh/GLI [6] are among the signaling pathways associated with PCa disease progression.

PI3K/AKT/mTOR pathway participates in multiple cellular functions including cell growth and proliferation [7]. Accordingly, different mutations regarding different PI3K/AKT/mTOR pathway components have been encountered in about 42% and 100% of primary and metastatic prostate tumors, respectively [8].

Hh/GLI pathway plays important roles in embryonic development, organogenesis and in adult tissue repair and proliferation. Thus, aberrant activation of such pathway has been implicated in many cancers including PCa [6]. However, the only mutation in Hh/GLI pathway components suspected to be involved in the development and progression of PCa is the loss of function mutation of suppressor of fused homolog, the major repressor of GLI activity [9].

The crosstalk between PI3K/AKT/mTOR and Hh/GLI pathways was reported in many cancer types [10–12]. Therefore, assessing the antitumor effects of the combination of the two pathways antagonists in PCa cells are strongly recommended [6].

Dactolisib is a dual PI3K and mTORC1/2 inhibitor that inhibits p-AKT up-regulation through mTORC2 and so is expected to show antitumor activity against various human cancers including PCa than mTORC1 inhibitors. Glioma antagonist 61 (GANT61) is an inhibitor of the Hh signaling pathway. By blocking the GLI function, which constitutes the final step in the Hh pathway, GANT61 inhibits cell proliferation *in-vitro* in a GLI-dependent manner and sensitizes PCa cells to ionizing radiation [13].

Accordingly, our objective was to evaluate the potential antitumor effects of GANT61 and Dactolisib on PC3 cells and to investigate whether their combination could add therapeutic benefit over their single treatments.

## Materials and methods

### Drugs under study

Dactolisib and GANT61 (Selleck Chemicals, Houston, Texas, USA) were prepared in a stock concentration of 10 mM using dimethyl sulphoxide and stored at —20 °C.

### In-vitro model of PCa

Human PC3 prostate cancer cells obtained from the American Type Culture Collection (ATCC, Manassas, Virginia, USA), were cultured in Dulbecco’s Modified Eagle’s Medium (DMEM) (Biowhittaker™, Lonza, Verviers, Belgium) supplemented with 10% fetal bovine serum (PAA, Brazil), 100 U/ml penicillin, and 100 μg/ml streptomycin (Lonza Verviers SPRL, Verviers, Belgium) at 37° C in humidified air containing 5% carbon dioxide.

### Experimental design

Three replicas of PC3 cells received either DMSO (vehicle), GANT61 (5 µM), Dactolisib (1.0 µM), or GANT61 (5 µM)/Dactolisib (1.0 µM) combination. All experimental procedures followed the regulatory aspects regarding the use of cell lines.

### Growth inhibition 50 (GI50) assay

Drugs cytotoxicity was determined by classic Microculture Tetrazolium Test (MTT assay) [14] where purple Formazan crystals are formed as a result of the reduction of the yellow tetrazolium ring by metabolically active cells. Formazan crystals have λ max at 540 nm and are directly proportional to the number of viable cells. Cells were plated at a concentration of 5000 cells/well in a 96-well microtiter plate and were then incubated at 37 °C in 5% CO_2_ for 24 h to allow cell attachment. The culture medium was then replaced with 200 µl treatment medium containing the drugs at different concentrations (Dactolisib: 0.125, 0.25, 0.5, 1, 2 and 4 µM), (GANT61: 0.625, 1.25, 2.5, 5, 10 and 20 µM) and the plate was then incubated at the same conditions for 72 h. CompuSyn software version 1.0 was used to calculate the GI50 of Dactolisib and GANT61 in PC3 cells.

### Biochemical analyses

For the determination of active caspase-3 levels in different cell lysates, caspase-3 colorimetric assay kit (Sigma Aldrich, Saint Louis, Missouri, USA) (Cat #: CASP-3-C) was used. Vascular endothelial growth factor 1 (VEGF1), cyclin-D1, protein kinase B (p-AKT), p70 ribosomal s6 protein kinase 1 (pS6K1), and Microtubule-associated proteins 1A/1B light chain 3 (LC3) were determined using the following ELIA kits (Cusabio, Houston, Texas, USA) (Cat #:CSB-E11718h), (LifeSpan BioSciences, Inc., Washington, USA) (Cat #: LS- F4095), (RayBiotech, Norcross, Georgia, USA) (Cat #PEL-AKT-S473-T), (Abcam, Cambridge, Massachusetts, USA) (Cat #ab176651), (Aviva Systems Biology, San Diego, California, USA) (Cat #OKEH01675), respectively according to the manufacturer’s instructions.

### *GLI1 *gene expression analysis using quantitative real-time PCR

Total RNA was extracted using Easy-spin™ total RNA extraction kit (Intron Biotechnology, Seongnam, South Korea) (Cat #: 17,221) followed by the determination of RNA quantity and purity using Nanodrop 2000 spectrophotometer (Thermo Fischer Scientific, Waltham, Massachusetts, USA). qRT-PCR was performed using the SensiFast™ SYBR® No-ROX one-step kit (Bioline Co., London, UK) (Cat #: BIO-72001). The sequences of the forward and reverse primers for *GLI1* gene were F: 5′- TTCCTACCAGAGTCCCAAGT-3′ and R: 5′-CCCTATGTGAAGCCCTATTT-3′, whereas those for the housekeeping gene (*β-actin)* were F: 5′-CTGGAACGGTGAAGGTGACA-3′ and R: 5′-AAGGGACTTCCTGTAACAATGCA-3′ [15, 16]. To confirm the expected unique amplification of *GLI1* and *β-actin* genes, the sequences of the primers were blasted against NCBI/Primer Blast. The analyses were performed as triplicates. The relative expression level of *GLI1* gene against *β-actin* as a housekeeping gene depended on ∆∆CT method.

### Statistical analysis of the data

Data was expressed as means ± standard error of the mean (SEM). Multiple comparisons were done using one-way analysis of variance (ANOVA) followed by the post-hoc test and the P-value < 0.05 was accepted as the level of significance. All statistical tests and figures were carried out using Graph Pad Prism® software package, version 6 (GraphPad Software Inc., San Diego, California, USA).

## Results

### Determination of GI50 of Dactolisib and GANT61 in PC3 cells

Both Dactolisib and GANT61 have shown dose-dependent cytotoxic effects on PC3 cells. Firstly, the exposure of PC3 cells to Dactolisib in the dose range of (0.125–4 µM) has exhibited linear dose–response curve with a GI50 of 1 µM. Secondly, the GI50 for GANT61 was found to be 5 µM as obtained from the dose–response curve of PC3 cells upon exposure to such drug at the dose range of (0.625–20 µM).

### Effect of different treatments on p-AKT and pS6K1 protein levels in PC3 cells

**Fig. 1:**
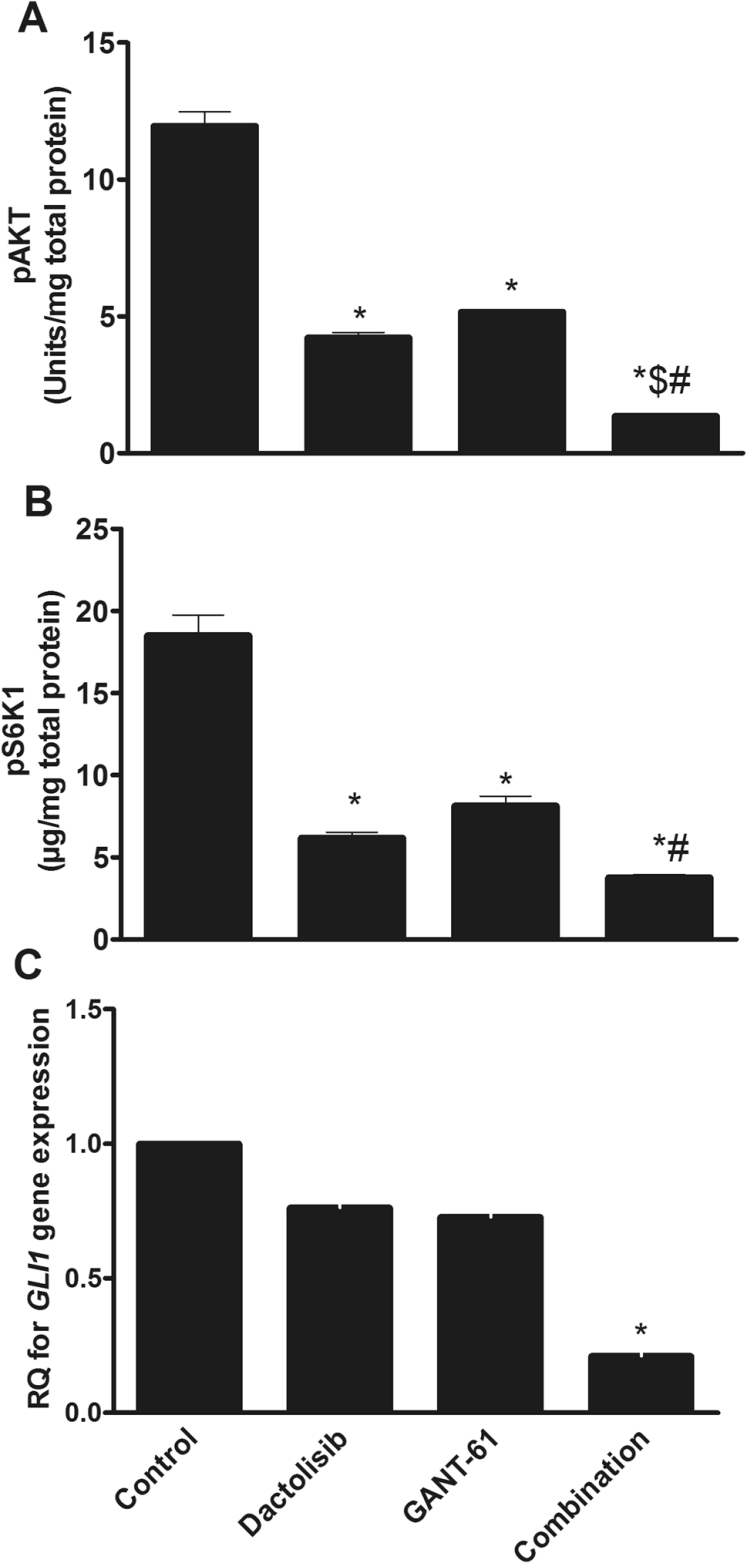
Effect of Dactolisib (1 µM) GANT61 (5 µM) and Dactolisib (1 µM)/GANT61 (5 µM) combination on **A** p-AKT protein level **B** pS6K1 protein level and **C** *GLI1* gene expression in PC3 cells PC3 cells were treated with Dactolisib (1 µM) and/or GANT61 (5 µM) then harvested after 72 h *GLI1* gene expression was determined by qRT-PCR while p-AKT and pS6K1 proteins’ levels were determined by ELISA technique as mentioned previously in the Materials and Methods section Data are expressed as means ± SEM * indicates *p* < 0.05 vs control $ indicates *p* < 0.05 vs Dactolisib-treated group and # indicates *p* < 0.05 vs GANT61-treated group

The results presented in Fig. 1A inferred that p-AKT protein levels were significantly reduced by 64.7%, 56.8%, and 88.6% in Dactolisib, GANT61, and combination-treated cells respectively (*p* < 0.001) compared to the control group. Moreover, the reduction in p-AKT levels induced by the combination treatment differs significantly (*p* < 0.01) from single treatments with either Dactolisib or GANT61. Likewise, pS6K1 levels were significantly reduced in Dactolisib, GANT61 and combination-treated groups by about three-folds (*p* < 0.001), 2-folds (*p* < 0.01) and five-folds (*p* < 0.001), respectively when compared to the control group as demonstrated in Fig. 1B.

### Effect of different treatments on *GLI1* gene expression in PC3 cells

Results shown in Fig. 1C revealed a profound down-regulation in *GLI1* gene expression in the three treatment groups compared to the control group. Dactolisib, GANT61 and their combination suppressed *GLI1* gene expression by about 23.8%, 27.3%, and 78.9%, respectively. However, the only statistically significant reduction in *GLI1* gene expression was achieved by the combination treatment (*p* < 0.05).

### Effect of different treatments on the cell death biomarkers (active caspase-3 and LC3) in PC3 cells

**Fig. 2:**
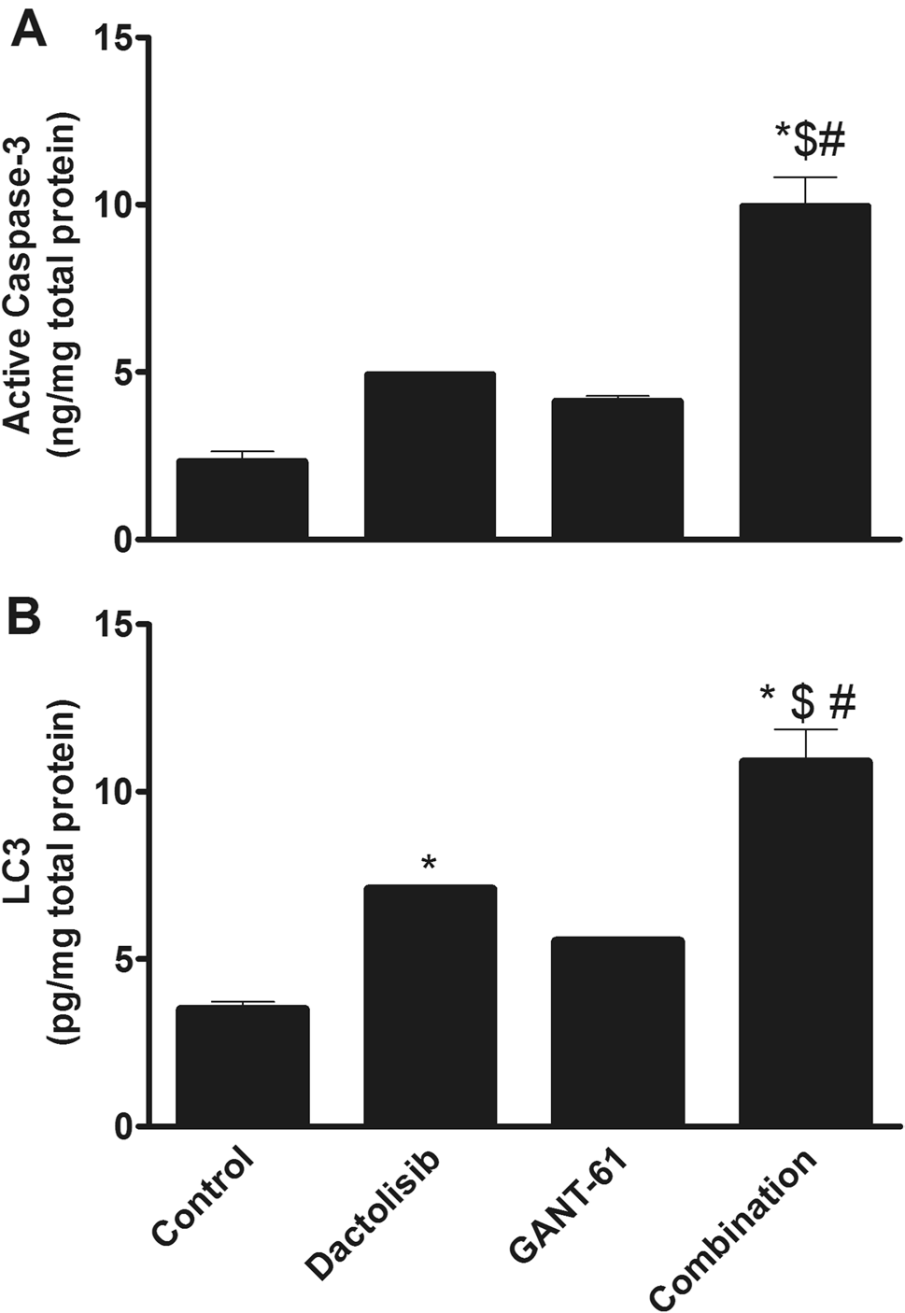
Effect of Dactolisib (1 µM) GANT61 (5 µM) and Dactolisib (1 µM)/GANT61 (5 µM) combination on **A** active caspase-3 protein level and **B** LC3 protein level in PC3 cells PC3 cells were treated with Dactolisib (1 µM) and/or GANT61 (5 µM) then harvested after 72 h Active caspase-3 level was determined colorimetrically while LC3 protein level was determined by ELISA technique as mentioned previously in the Materials and Methods section Data are expressed as means ± SEM * indicates *p* < 0.05 vs control $ indicates *p* < 0.05 vs Dactolisib-treated group and # indicates *p* < 0.05 vs GANT61-treated group

As depicted in Fig. 2A, treatment of PC3 cells with Dactolisib, GANT61 and their combination led to the over-activation of caspase-3 in comparison with the control group. Additionally, it was found that only Dactolisib/GANT61 combination treatment induced a statistically significant difference in caspase-3 activity when compared with the control group (*p* < 0.01).

Interestingly, Dactolisib-treated cells have shown up 2-folds increase in LC3 protein level in comparison with the control group (*p* < 0.05). Moreover, Dactolisib/GANT61 combination-treated cells have shown up statistically significant increase in LC3 level when compared with the control group (three-folds increase) (*p* < 0.01), Dactolisib-treated cells (1.5-folds increase) (*p* < 0.05) and GANT61-treated cells (two-folds increase) (*p* < 0.01) as depicted in Fig. 2B.

### Effect of different treatments on the cell cycle progression biomarker (cyclin-D1) in PC3 cells

**Fig. 3:**
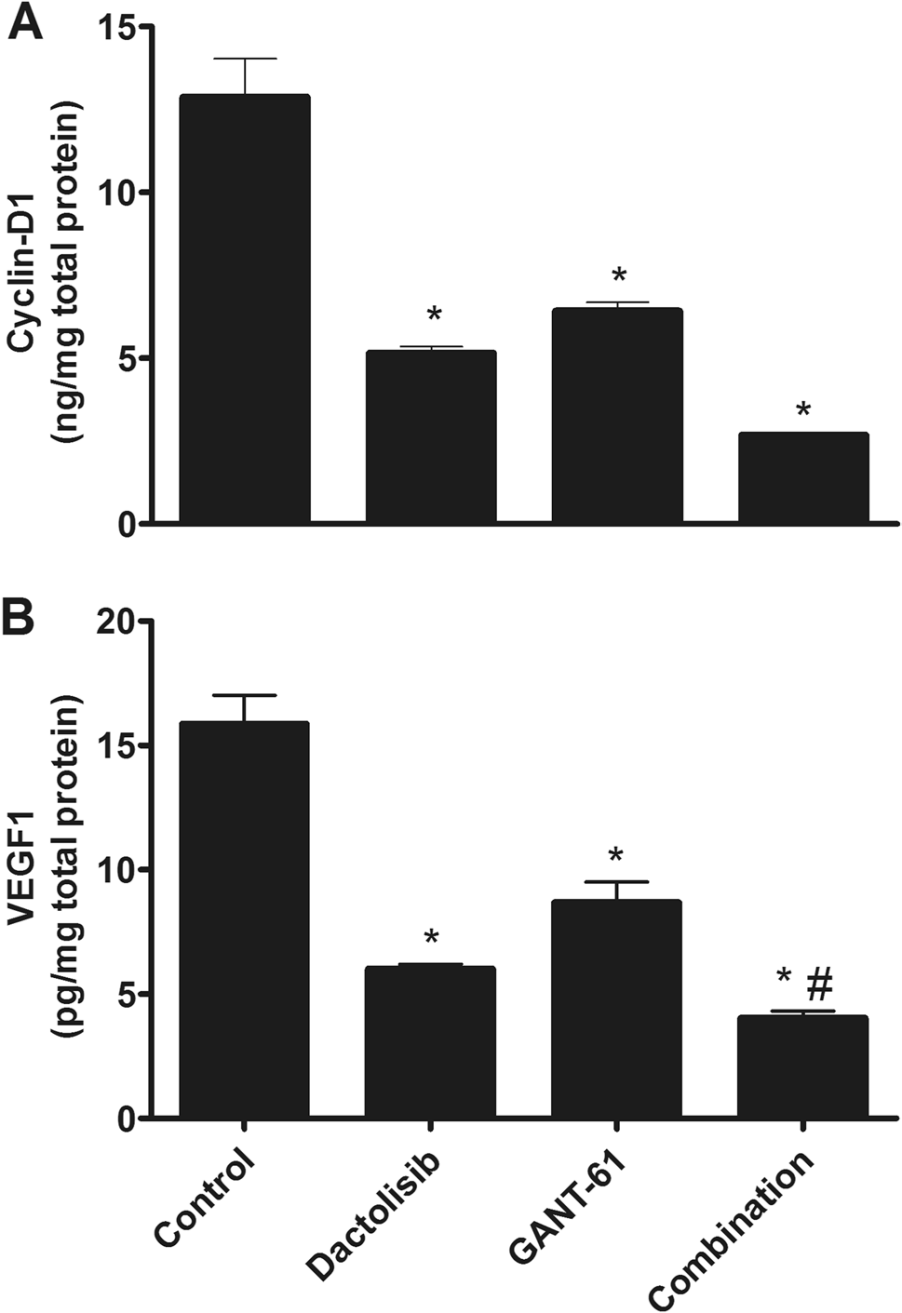
Effect of Dactolisib (1 µM) GANT61 (5 µM) and Dactolisib (1 µM)/GANT61 (5 µM) combination on **A** cyclin-D1 protein level and **B** VEGF1 protein level in PC3 cells PC3 cells were treated with Dactolisib (1 µM) and/or GANT61 (5 µM) then harvested after 72 h Cyclin-D1 and VEGF1 proteins’ levels were determined by ELISA technique as mentioned previously in the Materials and Methods section Data are expressed as means ± SEM * indicates *p* < 0.05 vs control $ indicates *p* < 0.05 vs Dactolisib-treated group and # indicates *p* < 0.05 vs GANT61-treated group

Results shown in Fig. 3A demonstrated that treatment of PC3 cells with Dactolisib or GANT61 as single treatments significantly decreased cyclin-D1 protein levels by about 60% (*p* < 0.01) and 50% (*p* < 0.01), respectively in comparison with the control group. Furthermore, Dactolisib/GANT61 combination significantly reduced cyclin-D1 level by about 80% in comparison with the control group (*p* < 0.001).

### Effect of different treatments on the angiogenic biomarker (VEGF1) in PC3 cells

Our results shown here in Fig. 3B inferred that both drugs and their combination reduced VEGF1 levels by about 62%, 45% and 75%, respectively compared to the control group (*p* < 0.01).

## Discussion

Development and progression of PCa to the most aggressive state mCRPC makes prostate cancer cells more resistant to approved chemotherapies (Docetaxel and Cabazitaxel) [3]. Many theories have evolved to elucidate mechanisms of such resistance. Abnormal activation of multiple signaling pathways has been described as one of the well-studied mechanisms for mCRPC progression despite continual hormonal therapy [17].

Several signaling pathways have been shown to be implicated in PCa disease progression. Among them, PI3K/AKT/mTOR and Hh/GLI pathways have been considered as two pathways that can participate either separately or through pathway crosstalk in mCRPC development [18]. In this regard, the current work aims to assess the antitumor effects resulting from the co-targeting of PI3K/AKT/mTOR and Hh/ GLI pathways in PC3 cells using Dactolisib and GANT61 as PI3K/AKT/mTOR and Hh/ GLI pathways inhibitors, respectively and also to investigate whether their combination add therapeutic benefit over their single treatments.

Pertaining to *GLI1* gene expression, it was found that Dactolisib, GANT61 and their combination down-regulated such expression level. As a target gene for GLI1 transcriptional factor [19], *GLI1* gene expression is normally down-regulated by GANT61. However, down-regulation of such gene expression by Dactolisib and to a better extent by GANT61/Dactolisib combination may support the existence of a non-canonical activation of Hh/GLI pathway by PI3K/AKT/mTOR pathway as reported by previous studies [20, 21]. Moreover, low p-AKT (a downstream kinase to PI3K [22]) and pS6K1 (a downstream kinase to mTORC1 [23]) protein levels have been observed due to Dactolisib, GANT61 and their combination treatment when compared with their levels that are found in vehicle control cells. This may clarify the capability of *GLI1* protein to activate PI3K/AKT/mTOR pathway through different mechanisms [18, 24].

Regarding the results of the effect of the treatment protocol on caspase-3 activity, both drugs were unable to initiate apoptotic cell death in PC3 cells when used individually while their combination induced apoptosis in PC3 cells. Our observation is concordant with the results of a previous study that has been conducted to elucidate the exact mechanism by which Dactolisib induced cell death in different PCa cell lines [25]. However, the inability of GANT61 to initiate apoptosis in PC3 cells has been opposed by the results of a study conducted to assess the effect of inhibition of *GLI1* on the proliferation of PC3 cells [26].

Concerning LC3 protein level, only Dactolisib but not GANT61 was able to initiate autophagy in PC3 cells. It was reported that Dactolisib can induce cell death in PC3 cells through initiation of autophagy as long as PC3 cells are *PTEN*-null in contrast to other PCa cell lines like DU145 which harbors wild-type PTEN [25]. However, the statistically significant increase in LC3 level in the combination-treated group compared to that in Dactolisib-treated group may indicate that GANT61 augmented Dactolisib in initiating autophagy in PC3 cells.

Considering the effect of the treatment protocol on cell cycle progression, the results demonstrated that both drugs and their combination have proven their ability to arrest cell cycle in PC3 cells. Taking into consideration the studies [27, 28] that have reported cyclin D1 as one of the downstream genes for GLI1 transcriptional factor, inhibition of *GLI1* by GANT61 could explain our finding regarding the suppression of Hh/ GLI pathway activity. Additionally, the cell cycle arrest that resulted from the dual inhibition of PI3K/mTOR by Dactolisib could be explained by the activation of glycogen synthase kinase 3 [29] which phosphorylates and promotes the degradation of cyclin D1 [30] and also by the inhibition of mTOR-related translational pathways resulting in the down-regulation of cyclin D1 protein expression [31].

Concerning the ability of PC3 cells for angiogenesis, treating cells with Dactolisib/GANT61 combination or with Dactolisib or GANT61 as single treatments has led to suppression of angiogenesis as documented by lower levels of VEGF1. Our results herein could definitely match the finding of another study that has reported *VEGF1* as one of the downstream genes for GLI1 transcriptional factor [9]. Therefore, inhibiting GLI1 activity by GANT61 has decreased the angiogenic potentiality of PC3 cells. Moreover, suppression of angiogenesis capability of PC3 cells after treatment with Dactolisib may be attributed to the reduction of the activity of Hypoxia-inducible factor 1 (HIF1), another transcriptional factor that controls the expression of *VEGF1* gene as described by a previous study that has clarified the role of PI3K and mTOR in the stabilization of HIF1α subunit [32].

To sum up, the present findings support the implication of PI3K/AKT/mTOR and Hh/GLI pathways and their crosstalk in PC3 cell cycle progression and in its ability for angiogenesis. Also, the current findings highlighted the possible additive/synergistic effect of Dactolisib and GANT61 in suppressing the viability of PC3 cells and in initiating programmed cell death through apoptosis and/or autophagy pathways. However, these findings still need further *in-vitro* and *in-vivo* studies to guarantee the applicability of implementing such drug combination in treating PCa.

## Data Availability

The data generated and analyzed during the current study are available from the corresponding author upon request.
